# A subwavelength resolution microwave/6.3 GHz camera based on a metamaterial absorber

**DOI:** 10.1038/srep40490

**Published:** 2017-01-10

**Authors:** Yunsong Xie, Xin Fan, Yunpeng Chen, Jeffrey D. Wilson, Rainee N. Simons, John Q. Xiao

**Affiliations:** 1 Department of Physics and Astronomy, University of Delaware, Newark, Delaware 19716, USA; 2 Department of Physics and Astronomy, University of Denver, Denver, Colorado 80208, USA; 3 Glenn Research Center, National Aeronautics and Space Administration, Cleveland, Ohio 44135, USA

## Abstract

The design, fabrication and characterization of a novel metamaterial absorber based camera with subwavelength spatial resolution are investigated. The proposed camera is featured with simple and lightweight design, easy portability, low cost, high resolution and sensitivity, and minimal image interference or distortion to the original field distribution. The imaging capability of the proposed camera was characterized in both near field and far field ranges. The experimental and simulated near field images both reveal that the camera produces qualitatively accurate images with negligible distortion to the original field distribution. The far field demonstration was done by coupling the designed camera with a microwave convex lens. The far field results further demonstrate that the camera can capture quantitatively accurate electromagnetic wave distribution in the diffraction limit. The proposed camera can be used in application such as non-destructive image and beam direction tracer.

Metamaterials are a class of artificial periodic structures with unit cell size much smaller than the electromagnetic wavelength of interest^1^. They can thus be treated as a continuous medium with electromagnetic response mostly controlled by their structures. Since the structures of the metamaterials can be properly designed with the assistance of simulation tools, the engineering of the electromagnetic responses for such medium has become very straightforward and efficient via such pathways. In recent years, unique electromagnetic properties such as invisible cloaking^2^,^3^, superlensing^4^,^5^, negative index^6^,^7^ have been both theoretically and experimentally demonstrated.

One impressive type of metamaterials is metamaterial absorber, which is designed to perfectly absorb the incident electromagnetic radiation in ideal situations. Due to their excellent properties, metamaterial absorbers have generated a great interest both in the scientific field, to fundamentally understand their properties, and in the engineering arena, to use such properties in creating or improving novel technologies^8^,^9^,^10^,^11^,^12^,^13^,^14^. One of the popular applications of metamaterial absorber is microwave cameras. The metamaterial absorber features with perfect absorption^15^,^16^,^17^,^18^,^19^, potentially high spatial resolution originated from its subwavelength unit cell size and wide working frequency from microwave to infrared with appropriate unit cell size and energy conversion sensor^14^. Therefore, the advantages of the metamaterial absorber based camera include but not limited to high signal sensitivityhigh spatial resolution, and minimal image interference or distortion to the original field distribution. Current Charge-coupled Device (CCD) camera has been well developed for optical imaging with subwavelength resolution^20^,^21^, while it is less applicable in frequency range such as terahertz and microwave, where the metamaterial based camera is ideal candidates^22^,^23^,^24^,^25^,^26^. Other than used as subwavelength resolution imaging camera, the metamaterial absorber based subwavelength camera has also found its value in applications such as non-destructive detection^27^, remote sensing^28^ and radio astronomy^29^.

To our knowledge, the only previous metamaterial absorber based device with imaging function (focal planar array) was constructed with complex microwave circuit^15^. In addition to the lack of demonstrations on imaging performance this design also has a few disadvantages, including high cost and difficulty in reaching high frequency range. Majority of the previous reported non-metamaterial absorber based microwave cameras require the microwave detection facility such as network analyzer^30^,^31^,^32^,^33^,^34^,^35^,^36^. Some of these systems further suffer from expensive, bulky and heavy system setup^30^,^31^,^32^,^33^ or low imaging speed because the detection demands mechanical movement^34^,^35^,^36^. This work reports the first metamaterial absorber based compact and portable subwavelength resolution camera. No mechanical movement is needed during the imaging measurement resulting from the high speed in scanning. The imaging capability of this camera is characterized in both near field and far field measurements. The near field characterization reveals that it produces qualitatively correct imaging result with negligible distortion to the original field distribution. The far field measurement further demonstrates that the camera captures quantitatively accurate image in the diffraction limit and can be used in application such as non-destructive image and beam direction trace.

## Theoretical model, simulation and experiment

**Figure 1:**
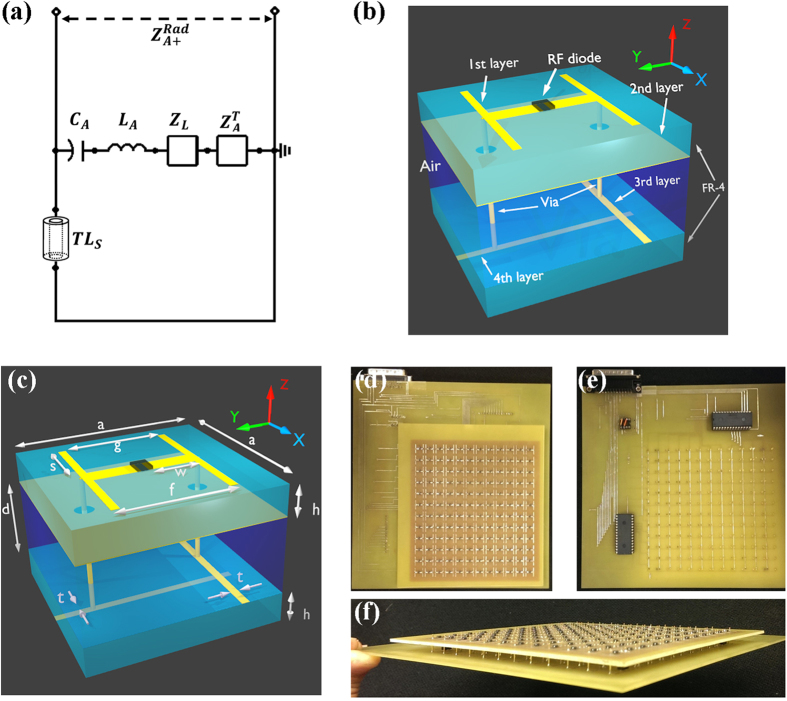
(**a**) The transmission line model of a metamaterial absorber. (**b**) Schematics and (**c**) structural dimension of the metamaterial absorber with detailed illustration on the parts indicated in the image and (**d**) top, (**e**) bottom and (**f**) side view of the fabricated metamaterial absorber camera.

Generally speaking, metamaterial absorber is consisted of two layers of periodic metal structures, termed as front and back metal structures hereafter, which are separated by a dielectric substrate. It has been both theoretically and experimentally proved that the electromagnetic behavior of a metamaterial absorber can be described by a transmission line mode^14^ as shown in Fig. 1(a). The front metal structure can be modeled as an RLC resonator, where L and C originated from the stored magnetic and electric field energy, respectively, R is used to describe the thermal energy generation. The substrate and metal back plane are modeled as a transmission line and a shorted line, respectively. The attached energy conversion sensor is modeled as an additional impedance component in series to the RLC resonator. This existing metamaterial absorber model indicates that none of the incident radiation wave will be reflected by selecting appropriate substrate thickness to match the impedance of the incident port, meaning all the input energy will be either converted into thermal energy or transferred to the energy conversion sensor^14^. Using this previous knowledge on metamaterial absorber as a basis, an experimental metamaterial absorber based subwavelength camera can be designed and analyzed by this model provided that the interaction between the nearby unit cells can be minimized and neglected.

The designed metamaterial absorber based subwavelength camera with energy conversion sensor is made of four layers of copper structures, the top two layers forms the metamaterial absorber and the bottom two is for DC scanning purpose. Each of metamaterial absorber layer comprises of an array of 12 × 12 unit cells with 1/5 of the working wavelength separated by a substrate made from a FR-4 board with thickness h = 1.57 mm and permittivity of *ε* = 4.4 − j0.088. In the first layer, there is an “H” shaped copper front structure with structural dimensional parameters of a = 10 mm, c = 8 mm, f = 6 mm, g = 5.2 mm, w = 2.2 mm and s = 3.5 mm, as shown in Fig. 1(b). The center of the “H” shaped is connected by a RF diode HSMS-286B, which is the energy conversion sensor, as shown in Fig. 1(b). The second layer has two 0.5 mm diameter through holes in each unit cell. The third and fourth layers are an orthogonal DC scanning network with linewidth of t = 0.4 mm and multiplexers shown in Fig. 1(d), locating at d = 4.5 mm underneath the second layer. The multiplexers are digitally controlled by an Elvis II DAQ card. Two vias, insulated to the copper back plates, bridges the first layer to the DC scanning network on the third and fourth layer.

To provide numerical characterization on the designed metamaterial absorber based subwavelength camera with energy conversion sensor, a numerical simulation was carried out using ANSYS HFSS. In the simulation, a single unit cell is defined as the structure shown in Fig. 1(b). To better illustrate the simulation setup, the “master and slave” function was used to simulate periodic boundary on the lateral surfaces, i.e. y-z and x-z surfaces in Fig. 1(b). The “radiation” function in the ANSYS HFSS was used to simulate vertical surface, i.e. x-y surface in Fig. 1(b). The energy conversion senor RF diode was simulated by a lumped port with frequency dependent impedance. More than 40,000 tetrahedron meshes in a single unit cell were adapted for calculation to obtain an accurate simulation result.

**Figure 2:**
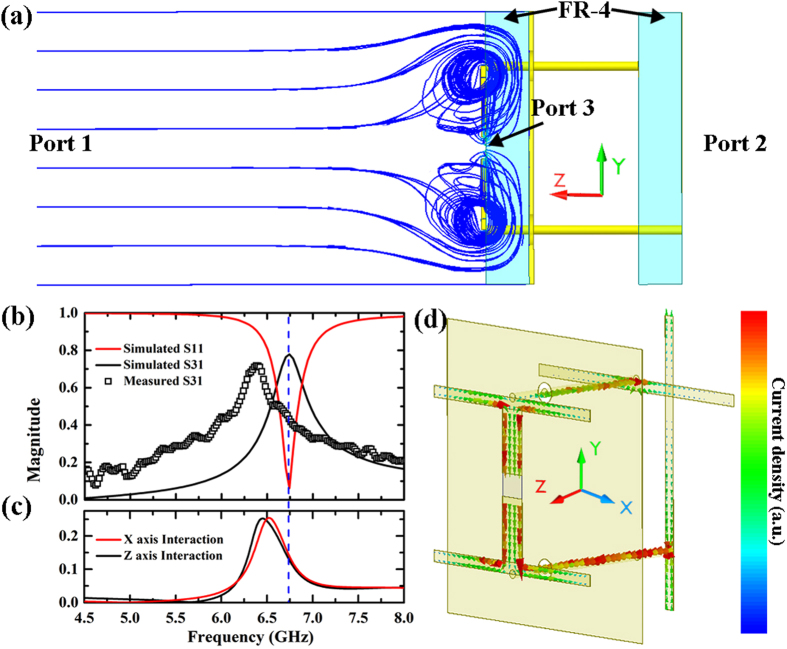
(**a**) Schematics of a single unit cell of metamaterial absorber based subwavelength camera with energy conversion sensor viewed along X axis. The simulated Poynting vector distribution is plotted in blue streamline. FR-4 substrate, Port 1, Port 2 and Port 3 are labeled in the figure. Port 3 is the RF diode. (**b**) Simulated S-parameters of the camera and measured transmission of the camera shown in legend. (**c**) Simulated transmission caused by the interaction between the nearby unit cells in both X and Y axis direction. (**d**) Simulated surface current density distribution on the 4 layers of copper metal structures.

To experimentally characterize the imaging capability and transmission intensity of the designed metamaterial absorber based subwavelength camera with energy conversion sensor, the following measuring set up was implemented. A linear polarized horn antenna was used to excite the metamaterial absorber array. The generated DC voltage on each unit cell, after going through a multiplexer-based selection circuit, was then detected by a digital source meter Keithley 2400. In previous conventional experiments, two ports should be accounted for in the metamaterial absorber setup: first port and second port are located at the front and back open space of the metamaterial absorber along the wave propagation direction respectively^8^,^18^. Simulation has shown that the above mentioned metamaterial absorber with energy conversion sensor has a maximum transmission between the first and second ports, abbreviated as S12 or S21, being about 2% (−33 dB), the maximum transmission between second and third ports, abbreviated as S23 or S32, being about 1% (−40 dB). Since the effect of port 2 is negligible, it is not going to be considered in either simulation or experiment measurements for this paper. In this paper, S31 is defined as the transmission measured from energy conversion sensor RF diode, port 3, towards the front open space, port 1, as shown in Fig. 2(a). The transmission was calculated by 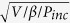, where *β* is the voltage sensitivity of the RF diode and *P*_*inc*_ is the incident microwave power in one unit cell. *P*_*inc*_ was calibrated using a microwave power meter and tapered slotline antenna with gain of 8 dB.

## Result and Discussion

The transmission measurement was carried out by feeding the excitation horn antenna with a continuous microwave with power of 0 dBm, the total microwave power shined on each imaging pixel was measured to be −43 dBm. In transmission measurements, the total energy could be consumed via three ways, i.e. reflection, transmission and absorption. At 6.75 GHz, a strong absorption with 6% (−24 dB) reflection was observed from simulation shown in Fig. 2(b). Because of the intrinsic lossy property of the FR-4 substrate, the simulated transmission (S31) is limited to be 78% (−2.2 dB). An experimental measured transmission is shown in the same figure, where the peak transmission was measured to be 72% (−2.9 dB) at 6.3 GHz. At least two reasons can be attributed to the frequency shift between the simulation and experimental demonstration. One of them could be impedance deviation of the diode in the measurement from its specification, since the impedance of the RF diode varies at different input power. The second reason could be the fabrication imperfections. The Poynting vector distribution at the maximum absorption frequency is plotted in Fig. 2(a). The incident microwave from port 1 (open space) is guided by the metal structure of the metamaterial absorber to be either trapped by the “H” front structure or fed into the port 2 (RF diode). The surface current density vector distribution on the metal structure is shown in Fig. 2(d). Strong current flow can be observed through the lumped port at the center of the “H” front structure and on the vias. The current distribution is similar to that of a dipole antenna^37^. The interaction between the nearby unit cells in both X and Y directions have also been simulated and shown in Fig. 2(c). The peaks of the interaction transmission are about 0.25 GHz lower than the perfect absorption frequency. The simulated interaction transmissions are both 0.15 (−16 dB) at the perfect absorption frequency (see blue dashed line). The interaction transmission between the nearby unit cell could not be measured in experimental conditions because (1) it is very hard to replace the diode with an port directly connector to the microwave network analyzer or microwave signal generator and (2) the diode cannot simply be replaced by a microwave excitation with the same complex impedance. However, the intensity of the interaction can be evaluated qualitatively by comparing the experimentally captured image results to the simulation, demonstrated in the following.

**Figure 3:**
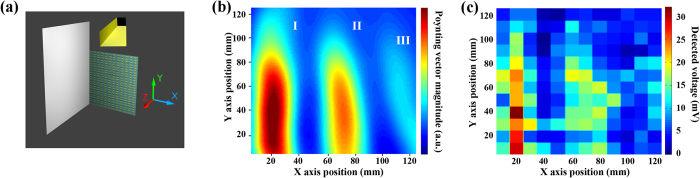
(**a**) The schematics of single plate interference pattern measurement, yellow object on top is simulated as the horn antennas, 140 mm × 140 mm gray sheet on y-z plane as aluminum plate, and the metamaterial absorber based subwavelength camera as the dielectric plate with 120 mm × 120 mm in lateral size, 20 mm in thickness with material parameters of *μ* = 1 and *ε* = 1 on X-Y plane. (**b**) The simulated single plate real Poynting vector magnitude distribution on Cartesian coordinates at. (**c**) The measured single plate voltage imaging results on Cartesian coordinates. The Cartesian coordinates range from 5 mm to 125 mm in both X and Y axises.

To test the near field imaging capability of the device, a series of experiments and simulations focusing on interference patterns generated from horn antenna and aluminum plate were examined. Two near field imaging setups were studied in details: single plate and double plates. Single-plate setup is shown in Fig. 3(a), where a 140 mm × 140 mm aluminum plate was vertically positioned with an angle of 15° to the Y-Z plane in order to introduce a tilted field distribution. In the simulation scheme, the metamaterial absorber based camera was modeled as a dielectric plate on the X-Z plane with 120 mm × 120 mm in lateral size, 20 mm thick in vacuum (i.e. completely transparent to microwave) to mimic the experimental setup. Both experiments and simulations were carried out at 6.3 GHz. For brevity and clarity, the 2D Poynting vector magnitude distribution were divided into three beam wave packets labeling (I), (II) and (III) on Cartesian coordinates, shown in Fig. 3(b). The simulated results from ANSYS HFSS show relatively high intensity at beam wave packets centering at (I) X = 20 mm, (II) 70 mm and (III) ranging from 110 mm to 120 mm. Both (II) and (III) appeared slight tilted resulting from the 15° incline towards X axis by the single plate. Compared to the simulated results, the experimental result (Fig. 3(c)) shows similar voltage distribution at estimated the same Cartesian coordinate positions. Such result provides the first indication that the metamaterial absorber based camera is able to map out the interference pattern of sinle plate setup. The camera behaves similarly to a vacuum plate, causing little distortion to the original field distribution. More importantly, it also provides accurate imaging result.

**Figure 4:**
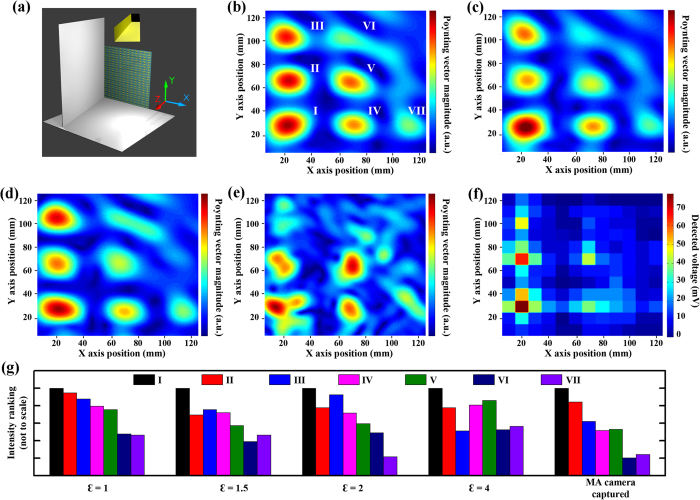
(**a**) The schematics of double plate interference pattern measurement, the yellow box on top is simulated as the horn antennas, 140 mm × 140 mm gray sheets on y-z plane and x-z plane as aluminum plates, and the metamaterial absorber based camera as the dielectric plate on x-y plane. The simulated real Poynting vector magnitude distribution on the camera with dielectric plate of 120 mm × 120 mm in lateral size, 20 mm in thickness, with material parameters of *μ* = 1 and *ε* = (**b**) 1, (**c**) 1.5, (**d**) 2 and (**e**) 4, (**f**) experimentally captured image with metamaterial absorber based camera. (**g**) Bar char of intensity rankings of the seven wave packets (**b**) to (**f**), where the Y axis of the bar chart represents the intensity ranking, which does not scale to the actual value. Larger value in Y axis describes higher intensity. The Cartesian coordinates range from 5 mm to 125 mm in both X and Y axises.

To further prove the accurate imaging and little distortion property of the metamaterial absorber based camera, a more complex interference pattern created by double aluminum plates was examined. Comparing to single plate, the interference pattern created by double aluminum plates is more complex and produces more variation when distortion is added. The experimental setup in the double-plate measurement is similar to the single plate measurement except that a second aluminum plate is added in the X-Z plane, as shown in Fig. 4(a). The 15° incline towards X axis of the vertically positioned plate is expected to introduce non-symmetric field distribution between X and Y axes. A series of simulations have been carried out to study the level of field distortion caused by the metamaterial absorber based microwave camera in near field measurement. In these simulations, the camera was again modeled by a dielectric plate with 120 mm × 120 mm in lateral size, 20 mm thick, permeability *μ* = 1, and permittivity *ε* = 1 (Fig. 4(b)), 1.5 (Fig. 4(c)), 2 (Fig. 4(d)) and 4 (Fig. 4(e)) respectively. According to these four simulation results, the level of field distortion systematically increases as the *ε* raises. At *ε* = 1 (Fig. 4(b)), 7 beam wave packets can be identified at (I) (20 mm, 20 mm), (II) (20 mm, 60 mm), (III) (20 mm, 100 mm), (IV) (70 mm, 20 mm), (V) (70 mm, 60 mm), (VI) (65 mm, 100 mm) and (VII) (110 mm, 20 mm) on the pointing vector distribution simulation result. The normalized intensity of the 7 wave packets have been summarized in Fig. 4(g).

We denote the intensity distribution observed in simulation from Fig. 4(b) as the original field, where we defined all the intensity distribution is un-distorted, and any intensity distribution deviating from Fig. 4(e) is recognized as distortion. Minor distortion in wave packet normalized intensity develops as *ε* begins to raises from 1 to 1.5 according to Fig. 4(g). As the distortion grows further, the intensity rankings keep evolving and finally the entire pattern deforms when *ε* reaches 4. The experimentally captured mostly resembles the simulation result with *ε* to be 1. Also comparing the un-distorted simulated Ponyting vector magnitude image Fig. 4(b) to the experimentally detected voltage image Fig. 4(f), (I) to (VI) wave packets can be found centering at the same Cartesian coordinate positions. However, wave packet (VII) was observed to be shifted by one pixel, which could be resulted from deviation between experimental and simulated setups due to possible bending of the aluminum plate and/or slight geometrical displacement of aluminum plates and horn antenna. Both single-plate and double-plate tests confirm that the metamaterial absorber camera is able to generate qualitatively accurate image in near field and produces minimal distortion to the original field during the measurement.

**Figure 5:**
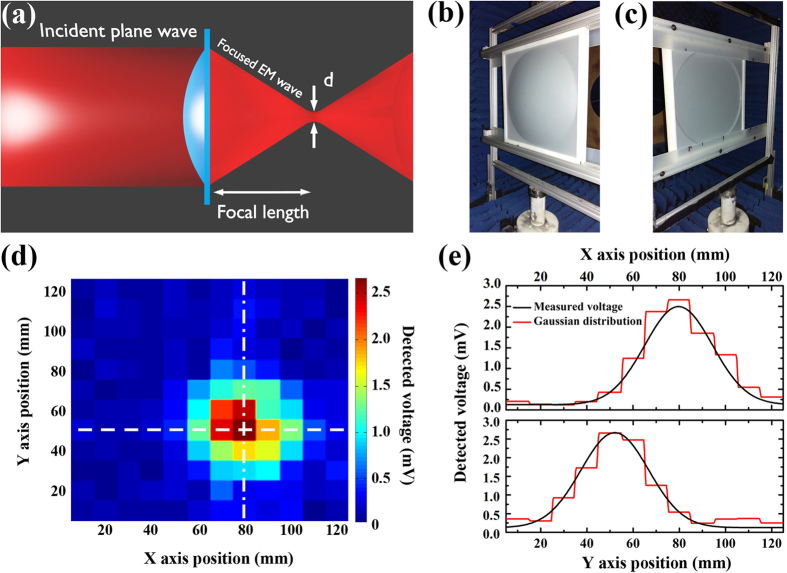
(**a**) The schematics of the setup of the lens in the beam focusing experiment. The electromagnetic wave (EM) and lens are depicted by red and blue color, respectively. The real life pictures of the microwave lens in the (**b**) front and (**c**) back. (**d**) 2D imaging result captured at the focal point of a convex microwave lens by the metamaterial absorber based camera. (**e**) Top panel: detected voltage at Y = 50 mm (indicated by white dashed line in (**d**)) is plotted against x-axis position in red solid line. Gaussian fitting result with parameter A mean of 79.7 mm and 95% confidence interval of (79.5 mm, 80.0 mm) is shown in black solid line. Bottom panel: detected voltage at X = 80 mm (indicated by white dashed-dot line in (**d**)) is plotted against x-axis position in red solid line. Gaussian fittings are the black lines with parameter B mean of 52.1 mm and 95% confidence interval of (51.9 mm, 52.3 mm). The measurement was carried out at frequency of 6.3 GHz.

To test the far field imaging capability of the camera, a microwave convex lens was used. This idea originates from literatures where the combination of an imaging sensor (CCD sensor) and a convex lens has been used a standard design for optical far field imaging devices^20^,^21^. In microwave range, such combined design has also been used in identifying the direction of a microwave source^38^,^39^,^40^. In this type of system, the directional sensitivity of the detection highly depends on the spatial resolution (i.e. the pixel size) of the microwave camera. The setup for this measurement has been illustrated in Fig. 5(a). Since the electromagnetic wave is well known to be one type of Gaussian beams, the intensity of the focused beam after the convex lens follows the Gaussian distribution. The full width at half maximum (FWHM) of the focused beam, labeled as “d” in Fig. 5(a), at the focal point of the lens should be about half of the wavelength^41^. The microwave lens used in our experiment has an aperture size of 29.5 mm and focal length of 12 mm. And the real life pictures of the lens can be found in Fig. 5(b,c).

Bear the Gaussian beam in mind, a quantitatively evaluation of the far field imaging capability of the camera can be obtained by fitting the captured voltage distribution to Gaussian equation shown in Eq. 1. Using the proposed imaging system, the 2D voltage distribution image captured at the focal point of the convex lens under frequency of 6.3 GHz is illustrated in Cartesian coordinates shown in Fig. 5(d). A squared 50 mm × 50 mm beam packet ranging from Y = 30 mm to Y = 70 mm, and X = 60 mm to X = 100 mm is observed with higher voltage intensity. From this beam package, largest voltage signal is detected at pixel X = 70 mm, Y = 40 mm, which is defined as the center of the beam. To obtain quantitative understanding on the far field imaging capability of the camera, a 2D Gaussian equation^42^.


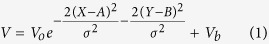


was used to fit the captured image. To better illustrate the fitting results, the detected voltage (red lines) and fitted data (black lines) are plotted in Fig. 5(e), top panel plots the voltage distribution at Y = 80 mm, and the bottom panel plots X = 50 mm. The detected voltage distribution well matches with fitted data. The fitted parameters and the corresponding descriptions have been tabulated in Table 1.

Although the center of the beam did not exactly locate at the center of a pixel, the location of the beam could still be well identified with very small uncertainty. The largest uncertainty at both axes is 0.4 mm, which is smaller than 1/100 of the wavelength. 1/*e*^2^ beam width of the beam has also been well recognized with a mean value of 29.3 mm, which is 61% of the wavelength in experimental measurements. This verifies that such measurement is carried out at the diffraction limit. Therefore, our camera not only has the capability of quantitatively mapping out the field distribution of the electromagnetic wave in both near field and far field, but also provides accurate quantitative information. This quantitative information could provide explicit data to help improve current understanding on subwavelength imaging system and applications such as non-destructive detection and beam directional trace^38^,^39^,^40^.

## Conclusion

The design, fabrication and characterization are reported for a novel metamaterial absorber based camera with subwavelength resolution. The camera features simplicity, portability, lightweight, high signal resolution and sensitivity, as well as minimal image interference or distortion to the original field distribution. For near field imaging, the experimental results are consistent with simulated interference patterns. The proposed camera features a spatial resolution of 10 mm at the detection wavelength of 48 mm and little distortion to the original field. For far field imaging, the 2D image data captured at the focal point of the microwave convex lens follows Gaussian beam distribution. Detailed quantitative information extracted from the fittings confirms that the proposed camera not only can quantitatively map out the field distribution of the electromagnetic wave in both near field and far field, but also provides accurate quantitative information on the imaging performance. Hence, in addition to providing experiment procedure for designing subwavelength imaging devices, this study also improves the current understanding on subwavelength imaging system and applications such as the non-destructive detection and beam directional tracer.

## Additional Information

**How to cite this article**: Xie, Y. *et al*. A subwavelength resolution microwave/6.3 GHz camera based on a metamaterial absorber. *Sci. Rep.* **7**, 40490; doi: 10.1038/srep40490 (2017).

**Publisher's note:** Springer Nature remains neutral with regard to jurisdictional claims in published maps and institutional affiliations.

## Figures and Tables

**Table 1:** Fitted result of Gaussian distribution beam with 2D image data captured at the focal point of an microwave convex lens.

Parameter	Unit	Fitted value	95% confidence intervals	Description
*V*_*o*_	mV	2.56	(2.52, 2.60)	Intensity of Gaussian beam
*V*_*b*_	mV	0.126	(0.121, 0.132)	Voltage background
*A*	mm	79.7	(79.5, 80.0)	X position of the beam center
*B*	mm	52.1	(51.9, 52.3)	Y position of the beam center
*σ*	mm	29.3	(29.0, 29.7)	1/e^2^ beam width
